# Principles of Zebrafish Nephron Segment Development

**DOI:** 10.3390/jdb11010014

**Published:** 2023-03-18

**Authors:** Thanh Khoa Nguyen, Madeline Petrikas, Brooke E. Chambers, Rebecca A. Wingert

**Affiliations:** Department of Biological Sciences, Center for Stem Cells and Regenerative Medicine, Center for Zebrafish Research, Boler-Parseghian Center for Rare and Neglected Diseases, Warren Center for Drug Discovery, University of Notre Dame, Notre Dame, IN 46556, USA

**Keywords:** nephron, segment, development, zebrafish, distal tubule, Tfap2a, Tfap2b, Kctd15

## Abstract

Nephrons are the functional units which comprise the kidney. Each nephron contains a number of physiologically unique populations of specialized epithelial cells that are organized into discrete domains known as segments. The principles of nephron segment development have been the subject of many studies in recent years. Understanding the mechanisms of nephrogenesis has enormous potential to expand our knowledge about the basis of congenital anomalies of the kidney and urinary tract (CAKUT), and to contribute to ongoing regenerative medicine efforts aimed at identifying renal repair mechanisms and generating replacement kidney tissue. The study of the zebrafish embryonic kidney, or pronephros, provides many opportunities to identify the genes and signaling pathways that control nephron segment development. Here, we describe recent advances of nephron segment patterning and differentiation in the zebrafish, with a focus on distal segment formation.

## 1. Introduction

### 1.1. Essential Functions of the Kidney

**Figure 1:**
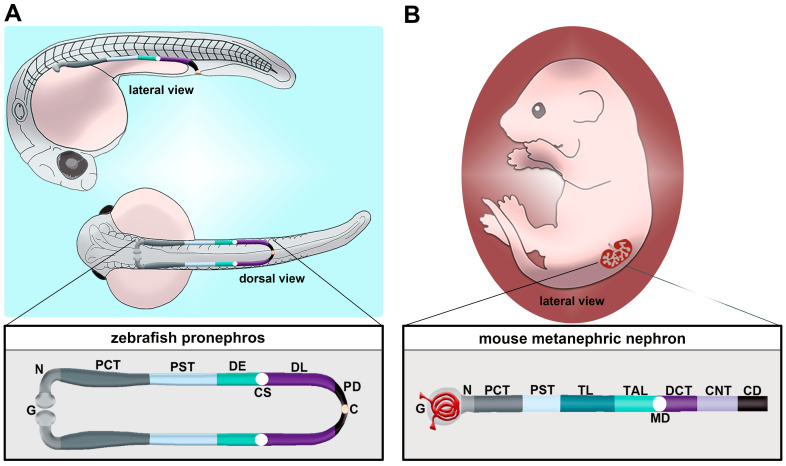
Comparison of nephron segment composition between the zebrafish and the mouse. (**A**) The zebrafish embryo forms a pronephros that consists of two nephrons located on either side of the midline, which form by the 24 h post fertilization (hpf) stage and quickly undergo morphogenesis events that enable them to begin blood filtration by approximately 48 hpf. (**B**) Nephron composition in the mouse metanephros; note that the nephron is drawn here in a linear configuration, but it would display a folded/convoluted anatomical configuration within the native kidney. There is a striking conservation of proximal and distal segments, but the thin limb is one notable distinction between these nephron forms. Abbreviations are as follows: P = podocytes; N = neck; PCT = proximal convoluted tubule; PST = proximal straight tubule; DE = distal early; CS = Corpuscle of Stannius; DL = distal late; CD = collecting duct; TL = thin limb; TAL = thick ascending limb; MD = macula densa; DCT = distal convoluted tubule; CNT = connecting tubule.

The kidney performs various vital functions in the body, such as removing metabolic waste products, monitoring blood pressure, secreting hormones and maintaining pH, electrolyte and water balance [1]. Nephron functional units in the kidney consist of subdomains, or segments, that are dedicated to particular physiological tasks (Figure 1) [2,3]. These segment regions include the renal corpuscle that filters the blood; tubule segments that modify the filtrate, including the proximal tubule, loop of Henle, distal tubule and connecting tubule; and lastly, the collecting duct, which performs the final modifications on urine and conveys it out of the kidney [4,5,6,7,8,9,10,11,12]. Within the renal corpuscle, the podocytes secrete collagen and growth factors to help maintain glomerular basement membrane and endothelial cell fenestration, respectively [4,5]. Additionally, the ultrastructural features of the podocytes are crucial components in establishing glomerular filtration [4,5]. The proximal tubule is important for reabsorbing nutrients, electrolytes and water: in total, it reabsorbs about two thirds of most water and important electrolytes, such as Na^+^, Cl^−^ and HCO3^−^, as well as 99.8% of glucose and amino acids filtered by the human kidney [6,7]. The loop of Henle is further divided into the descending thin limb (DTL), the ascending thin limb (ATL) and the thick ascending limb (TAL), which together play critical roles in water homeostasis [8]. This is achieved by the expression of the Na^+^/K^+^/2Cl^−^ cotransporter in the TAL, which helps the reabsorption of 25% of filtered Na^+^ [9]. Additionally, the TAL also reabsorbs filtered Ca^2+^ and Mg^2+^ [9]. The distal tubule is important in regulating electrolytes such as Na^+^, K^+^ and Ca^2+^ and pH levels [10]. The distal tubule reabsorbs approximately 5–10% of filtered Na^+^, as well as 7–10% of filtered calcium [10]. The collecting duct originates from the ureteric bud, and is essential in regulating water and electrolytes such as Na^+^ and Cl^−^ [11,12]. 

Given the crucial tasks accomplished by kidney nephrons, their proper formation is vital for normal renal function [13,14,15]. Alterations in the number and composition of nephrons have significant ramifications for kidney health, and can lead to congenital anomalies of the kidney and urinary tract (CAKUT) [16,17,18,19,20,21,22]. Understanding the mechanisms of nephrogenesis has many potential applications to treat both inherited and acquired renal diseases [23,24,25]. 

### 1.2. Vertebrate Kidney Forms Are Comprised of Nephrons

Several versions of the kidney organ are made and degraded during vertebrate ontogeny [26,27]. They develop in a prototypical sequence: the first form is termed the pronephros, the second form is termed the mesonephros and the third possible form is termed the metanephros. This last form is typically made in reptiles, birds and mammals. Across all forms, the nephron is the common structural and functional unit. In general, each version of the kidney in a given species exhibits increasing complexity with regard to the absolute number and arrangement of its nephrons. There is broad conservation of nephron segment composition. However, there are notable variations across the phylogenetic spectrum, such as the aglomerular nephrons in some species, and differences in whether nephrons possess a loop of Henle segment [23,28,29,30,31,32,33,34]. In this review, we specifically discuss kidney form and development in the zebrafish, *Danio rerio*, which has been used extensively over the past 25 years to study the mechanisms of nephron ontogeny, physiology and regeneration as well [35,36,37,38,39,40,41,42,43,44,45,46,47,48,49,50,51,52]. In the following sections, we provide an overview of the kidney forms, and then discuss the recent advances in understanding the genetic mechanisms of distal segment patterning and differentiation. 

## 2. Using the Zebrafish to Study Nephron Development

### 2.1. Zebrafish Pronephros Composition and Function

Unlike mammals, which manifest the pronephros, mesonephros and metanephros during development, the zebrafish only manifests the pronephros and the mesonephros [37]. Each of these kidney structures originates from populations of mesodermally derived renal progenitors. The zebrafish pronephros is extremely simple, being comprised of two nephrons that form rapidly and function throughout the first several weeks of life [53,54,55,56,57,58,59,60,61,62,63,64]. Key transcription factors such as Pax2a, Pax8, Hand1 and Osr1 have critical roles in the specification of the renal progenitor’s fate [55,65,66,67,68,69,70,71,72]. Once this mesenchymal identity is set, which is thought to transpire from the tailbud stage or 10 h post fertilization (hpf) through to 24 hpf, the renal progenitors undergo events that transition them to form tubules with defined proximal and distal epithelial cell identities [73,74,75,76,77,78,79,80,81,82,83,84,85,86,87,88,89,90]. The zebrafish segments include the neck (N), proximal convoluted tubule (PCT), proximal straight tubule (PST), distal early (DE) tubule and distal late (DL) tubule (Figure 1A) [73]. Compared to the typical mammalian nephron, the loop of Henle is missing (Figure 1B) [73]. At approximately 48 hpf, the pronephros will become functional and begin to filter the circulation [60]. Between 24 hpf and 48 hpf, there are several morphogenesis events which happen to create this functioning pronephros [37]. For example, podocyte precursors migrate to the midline and recruit blood vessels, forming the glomerulus. The tubular segments continue to differentiate, showing the expression of additional solute transporter genes that impart unique physiological capabilities to each segment. Combined with this is the growth of the renal tubule and coiling event of the PCT. 

### 2.2. Zebrafish Mesonephros Composition and Function: Spotlight on Renal Regeneration Studies

The adult form of the zebrafish kidney, also known as the mesonephros, develops beginning around 12–14 days post fertilization (dpf) [37,91,92]. During this process, more nephrons are created and joined with the existing pronephros tubules, forming a small collection of interconnected nephrons that drain via a pair of renal collecting ducts [37,91,92]. Eventually, this process generates between 300–500 nephrons [91,92]. The zebrafish body size positively correlates with the number of mesonephric nephrons [91,92]. After complete mesonephros generation, the zebrafish possesses more complex nephron branching, yet the segmental composition remains similar to that of the pronephros [92]. The adult mesonephros is ultimately located at the dorsal body wall surrounded by connective tissues. In the zebrafish, the mesonephros contains a head region, a saddle and a tail region. Additionally, based on the density of nephrons throughout the mesonephros, there are four further subdivisions: anterior nephron-dense region (ANDR), medial nephron-sparse region (MNSR), medial nephron-dense region (MNDR) and posterior nephron-sparse region (PNSR). Compared to mammalian kidneys, which cannot regenerate nephrons, in the zebrafish kidney, zebrafish keep adding nephrons to their kidney throughout their lifetime. In addition to nephron addition throughout development, the mesonephros can also regenerate damage to existing nephrons after injury [91,92,93,94,95,96,97,98,99,100,101,102].

## 3. Molecular Genetic Toolkit in the Zebrafish Animal Model

### 3.1. Zebrafish as a Model for Developmental Biology and Biomedical Research

The zebrafish offers a simple, yet effective, animal model to study developmental biology and biomedical research [103,104,105,106,107,108,109]. There are multiple traits that facilitate developmental research. First, the zebrafish has a simple architecture: at its embryonic stage, the embryonic kidney only comprises two nephrons [53]. Much due to their simplicity, zebrafish carry high similarity with mammalian kidneys: 70% of human genes share at least one homolog with the zebrafish [106]. The zebrafish kidney also shares most of the kidney segments with the mammalian kidney, with the exception of the mammalian loop of Henle [73]. Furthermore, zebrafish development occurs ex utero, and embryonic zebrafish are optically transparent, allowing for live imaging and visualization of organs. Next, zebrafish have high regeneration ability: new kidney nephrons keep being added to the developing kidney throughout the lifetime or after injury. Meanwhile, mammalian kidneys cannot regenerate or produce more nephrons after they are destroyed. Additionally, zebrafish demonstrate fast organogenesis, with the vital organs such as the kidney, heart and eyes available as early as 24 hpf. At 48 hpf, the kidney is fully functional, and all common organs are visible at 120–144 hpf. Coupled with this is high fecundity: zebrafish in a good laboratory environment can breed all year round, and each week they can breed up to 300 eggs. 

### 3.2. Forward Genetics: From Random Mutagenesis Screens to Chemical Screens

In forward genetic approaches, the screening of animal populations that have random mutations in the genome can uncover the novel importance of genes in biological pathways. To start this process, the first step is creating mutagenic lesions on the genome that are heritable and phenotypically distinguishable. There are several types of random mutagenesis which have been studied. Early work used gamma irradiation to create breaks in the chromosome to create mutant phenotypes. This method has a weakness as it causes large deletions, translocations or chromosomal aberrations that complicate the process to specify the genes responsible for the mutant phenotype. Subsequently, alkylating agents were used, such as ethylnitrosourea (ENU). In this process, chemical mutagens were exposed to adult males, causing random point mutations in their DNA [53]. The adult males were then bred with WT females, generating heterozygous F1. To expand heterozygous populations, F1 heterozygotes were then outcrossed again with WT to generate F2 heterozygous generation. F2 heterozygotes were then incrossed with each other to generate the desired homozygous mutants. This method provides more advantages over gamma irradiation, such as high mutagenic loads in the zebrafish genome, as well as the induced phenotypes being specifically linked to one gene. A major drawback of this method is that it is difficult to identify specific mutations responsible for the induced mutant phenotype. To overcome this challenge, several groups have introduced replication-deficient retroviruses or transposons as mutagens, which can insert into the genome to cause mutations. In this method, the mutagenic agent is inserted into the 1-cell stage if it is a transposon, or the 1000-cell stage embryos if it is a retrovirus. However, the overall mutagenic frequency from retroviruses and transposons has been estimated to be much lower than ENU, thus requiring a significantly more bountiful library of mutagenized fish. Both random mutagenesis and insertional mutagenesis methods have led to the generation of mutant collections with defects in kidney development [53,77,90,110,111,112,113]. Within the zebrafish pronephros, examples of aberrant morphological defects that could be attributed to reduced kidney function include pericardial edema, kidney cysts, hydrocephalus of the brain or body curvature [53,77]. 

Since the early live screens, scientists have expanded the methods of identifying mutant features. One simple way of phenotypic screening is by using a molecular marker(s) to visualize a particular cell or tissue type, using techniques such as whole mount in situ hybridization (WISH) or immunostaining [111]. The use of a tissue-specific transgenic line marked by fluorescent protein expression can alleviate the hands-on effort and sample fixation issue caused by in situ hybridization or immunostaining. In addition, restriction enzymes could offer a simple and useful tool to detect mutation. If the mutation affects a particular site on the DNA that a restriction enzyme has been known to affect, such as the removal of a restriction enzyme site from WT embryos, a restriction enzyme digest assay could be used to distinguish between WT and mutant embryos. Lastly, DNA extracted from embryos from mutant lines could be used for PCR and sent for sequencing, allowing for robust and direct identification of the genetic lesion. 

### 3.3. Reverse Genetics: Loss-of-Function Methods Using Morpholinos and Genome Editing

**Figure 2:**
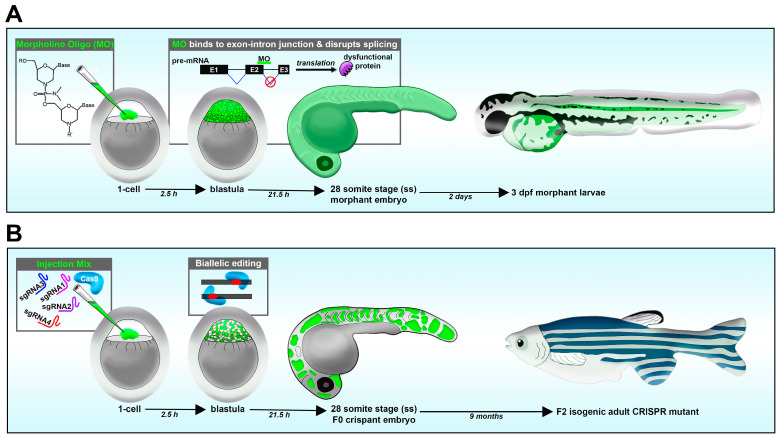
Reverse genetic loss-of-function strategies in the zebrafish embryo. (**A**) Morpholino knockdown. (**B**) CRISPR-Cas9.

Loss-of-function studies have been performed using various approaches, including morpholino oligos, CRISPR/Cas9 system, TALENs and ZFNs [46,108]. In comparison, techniques with RNA interference (RNAi) have proven to be problematic in zebrafish [114]. Morpholinos are a short antisense oligonucleotide, up to 25 basepairs (bps) long, designed to target the processed mRNA or pre-mRNA transcript of the animal [115]. Morpholino oligos that target the ATG start site block translation, while morpholinos that target the splice donor or acceptor site of a given exon–intron boundary can disrupt the spliceosome [115]. This can lead to numerous outcomes, such as introns being present after RNA processing. In such a case, a splice site morpholino might cause a frameshift mutation, resulting in a premature stop codon during translation, causing a truncated protein that will later be degraded. A benefit of splice-site morpholino over ATG-site morpholino is that RNA transcripts can be reverse transcribed into DNA, and the presence of the mis-spliced transcripts can be detected using RT-PCR and sequencing [115,116]. In the zebrafish model, morpholinos are typically delivered to the 1-cell-stage embryo via microinjection, and effects last for up to several days (1–3, or more), allowing for an easy model to study development (Figure 2A) [115]. 

Other loss-of-function methods which have been used in the zebrafish include zinc finger nucleases (ZFNs) [117,118,119,120] and transcription-activator-like (TAL) effector nucleases (TALENs) [121,122,123,124]. ZFNs were the earliest tool of genome-editing with the use of endonucleases [117,118,119,120]. They contain chimeric enzymes with a zinc finger (ZF) domain fused with the FokI endonuclease domain. The ZF domain mediates the binding to DNA, while the FokI endonuclease domain performs the nucleic acid cleavage. ZF domains can be engineered to bind to a 9–18 bp DNA sequence, and once that binding happens, the FokI domain causes a double-stranded break in the DNA, which is then repaired by either homologous cell repair or non-homologous end joining. Similarly to ZFNs, TALENs contain chimeric proteins with a structure containing a modular DNA-binding domain that is fused together with a FokI nuclease, causing a double-stranded break at a specific site in the gene of interest [121,122,123,124]. TALENs originate from virulence factors in *Xanthomonas* bacteria. The difference between TALENs and ZFNs are that DNA-binding modules of TALENs are naturally occurring, termed TALs. TALs include a series of conserved domains across 34 amino acids, with the only difference being between Positions 12 and 13, where DNA contact sites happen, termed repeat variable di-residues. 

In recent years, the CRISPR/Cas9 system has become the most popular tool to study gene knockout in the zebrafish [123,124]. CRISPR stands for “clustered regularly interspaced short palindromic repeats”, which are segments of prokaryotic DNA that bacteria use to defend against foreign DNA elements by binding to these sequences and recruiting the CRISPR-associated protein 9 (Cas9) to destroy such a DNA sequence [125]. Interestingly, researchers can utilize this ancient self-defense mechanism to edit the genome of animal models such as the zebrafish [126,127,128,129]. In this design, a guide RNA (gRNA) can be designed to bind specifically to a gene of interest which will recruit a Cas9 endonuclease, which will make a double-strand cut in the DNA sequence. This cut will thus result in the generation of a random insertion/deletion (indel) mutation in the genome as a result of non-homologous end joining. The introduction of specific mutations can also be performed by including single strand oligos with the guide RNA and Cas9 mixture [130]. CRISPR/Cas9 engineered embryos can later be extracted for DNA for sequencing to detect the genetic alterations. Additionally, the T7 endonuclease assay can be used to detect mutations caused by CRISPR/Cas9. In this assay, T7 endonuclease will recognize deformities in heteroduplex DNA and make a cleavage, as compared to WT samples. The results could be easily detected via an agarose gel, making the T7 endonuclease assay a cost-effective and simple way of verifying the CRISPR reaction. 

Similar to morpholino oligos, in CRISPR/Cas9, both the gRNA and Cas9 enzyme are delivered into zebrafish embryos in the 1-cell stage via microinjection. Further, a cocktail of multiple gRNAs can be simultaneously injected with the Cas9 to increase the probability of mutation, and to introduce biallelic disruptions and thus permit analysis in the F0 embryos for a gene of interest (Figure 2B). CRISPR/Cas9 differs from morpholino oligos in that the target of disruption is DNA instead of RNA. Therefore, CRISPR/Cas9 can serve as a powerful method to generate knockout or knock-in alleles depending on the materials which are injected. Resulting crispants (F0) can be raised and heterozygotes can be outcrossed with WT to generate F1 generation, consisting of ~50% WT and ~50% heterozygotes. Selected F1 heterozygotes can then be incrossed with each other to generate F2 progeny with 25% homozygous for mutation. One caveat is that gene targeting during the initial 1-cell microinjection can lead to genome modifications in later cleavage cycles, such as in the 2- or 4-cell stages, and thus individual embryos can be mosaics of cells with multiple different mutations. Thus, when raising an F1 generation, each individual may have varying mutations and detailed analysis is necessary when finding stable lines. There have been continuing advances that reduce off-target modifications to the genome when employing CRISPR/Cas9, such as the development of high-fidelity Cas9 derivatives [131].

It is highly desirable to study genetic lesions induced by CRISPR/Cas9 or other modes of gene editing, as studies have suggested that there are wider off-target effects of agents such as morpholinos that contribute to morphant phenotypes [132]. However, one downside of crispants or stable mutant lines generated via CRISPR/Cas9 is that milder phenotypes in many cases have been found to be the result of compensatory mechanisms where other genes are upregulated in response to the loss-of-function [133]. Thus, careful controls such as rescue studies and gene expression analyses are necessary to best validate each model and understand its potential research limitations [134].

### 3.4. Reverse Genetics: Gain-of-Function Approaches with mRNA and Transgenic Models

**Figure 3:**
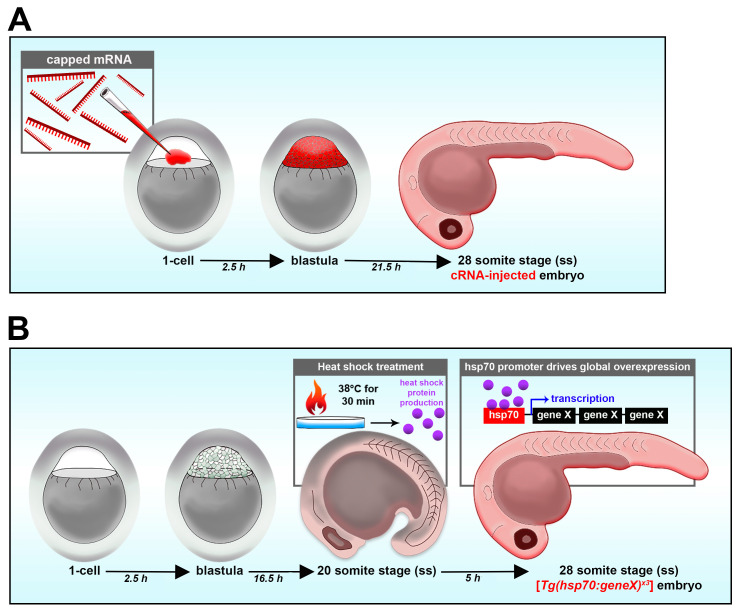
Reverse genetic gain-of-function strategies in the zebrafish embryo. (**A**) Overexpression through mRNA delivery. (**B**) Transgenic mediated overexpression.

In addition to loss-of-function studies, gain-of-function studies also allow researchers to properly study how certain genes influence development [46,108]. Gain-of-function can be achieved by transient overexpression via mRNA or transgenic heat shock [114]. Gain-of-function studies are helpful to study how the overexpression of a gene of interest affects nephron development, or to perform rescue studies in loss-of-function models and test the specificity. The microinjection of synthetic 5′ capped mRNA (cRNA) (as opposed to the delivery of plasmid DNA) leads to a relatively homogenous distribution of the mRNA and resultant protein throughout the developing embryo when performed in the zebrafish embryo in the 1-cell stage (Figure 3A) [114]. cRNA can also be co-injected with other molecules such as morpholino oligos to rescue nephron segment-specific phenotypes caused by morpholino oligos. Embryos could then be fixed at the time point of interest along with WT embryos or morphant embryos and utilized for further analysis of nephron segments such as whole mount in situ hybridization (WISH). While cRNA injection into embryos has shown success in overexpression or rescue from loss-of-function studies, as a transient method, cRNA injection might not successfully rescue nephron segment defects due to dosage, or may lead to disruptions in other developmental events [114]. 

In such cases, the use of a heat shock transgenic line is an alternative conditional strategy to achieve gain-of-function [114,135]. This could be achieved by creating a transgenic line with the Hsp70 promoter tagged to the gene of interest, called [*Tg(hsp70:geneX)*]. The transgenic line could be generated via the use of the Tol2 transposase system, which has been regarded as a powerful method to generate transgenic lines. This is achieved by injecting a construct including the Tol2 vector Hsp70 coupled with the gene of interest together with the Tol2 transposase mRNA into the one-cell-stage embryos. The Tol2 transposase will help to insert the construct into the genome of the embryos. Once the transgenic line is established, their embryos could be used for gain-of-function studies. To activate the transgene, heat shock treatment would be applied to the embryo at the desired time point of interest, such as 37 °C for 30 min in the 20 somite stage (Figure 3B). The heat shock treatment would promote the overexpression of the gene of interest for gain-of-function or rescue studies [135]. 

## 4. Advances in Understanding Distal Segment Development

Through a combination of forward genetic screens and reverse genetic approaches, zebrafish researchers have uncovered many key principles of pronephros development over the past 25 years. Seminal forward screens identified crucial transcription factors such as *pax2a* and *hnf1ba* [53,110]. The conducting of large-scale gene expression analysis screens and subsequent deposition of these data into public databases such as the Zebrafish Information Network (ZFIN, https://zfin.org/, accessed on 1 January 2023) [136,137] provided a wealth of information regarding the putative candidate genes involved in organogenesis, including the kidney. Screens for morphological signs of renal dysfunction such as edema have also been performed and used to identify the relevant kidney genes [77,90]. Subsequent targeted screens that directly assessed the presence of nephron segment populations with molecular markers have also successfully uncovered relevant genetic factors and signaling pathways. The mechanisms that regulate specification and early pattern formation of renal progenitors have been nicely reviewed elsewhere [138]. In the following sections, we will explore several recently published studies that used the study of selected candidates and/or the characterization and cloning of mutants from nephron segment screens to add new insights into our understanding about the development of distal segments, with a focus on the DE, DL and CS cell lineages. 

### 4.1. The Mecom/Tbx2a/2b/Emx1 Network in DL Pronephros Development

Genetic studies from several reports have revealed a set of transcription factors that are necessary for promoting the DL lineage. The *mds1/evi1 complex* (*mecom*) transcription factor was the earliest known marker of distal tubule progenitors [73,77]. Interestingly, in 2014, Li et al. reported that the knockdown of *mecom* leads to a reduced DL segment length [139]. In conjunction, they found that the proximal tubule (including both the PCT and PST segments) expands caudally [139]. Several years later, Drummond et al. identified roles for the *t-box2a* (*tbx2a*) and *t-box2b* (*tbx2b*) transcription factors in DL development as well [140]. The researchers found that the single knockdown of *tbx2a* or *tbx2b* led to the formation of a small DL segment, similar to the *tbx2b* genetic mutant, *from beyond*, or *fby^c144^*, which encodes a point mutation that introduces a premature stop codon within the T-box DNA-binding domain [140,141]. Embryos that were doubly deficient for *tbx2a*/*b* displayed a similar phenotype to each of the single gene knockdowns, suggesting the possibility that they act within the same pathway to influence the DL’s fate [140]. Subsequent research by Morales et al. in 2019 determined that *mecom* acts upstream of *tbx2a*/*b,* finding that *tbx2b* overexpression was sufficient to rescue *mecom*-deficient embryos [142]. Furthermore, they identified that the transcription factor *empty spiracles homeobox gene 1* (*emx1*) was highly expressed in distal tubule progenitors [142]. The domain of *emx1+* distal tubule progenitors was reduced in both *tbx2a*- and *tbx2b*-deficient embryos, and *emx1* was sufficient to rescue DL formation in the latter [142]. These and other studies enabled the authors to propose a working model for DL fate in which Mecom promotes *tbx2a/b* expression, and Tbx2a/b in turn promotes *emx1* expression. Future studies with genetic mutants will be a useful way to test this model further. Additionally, transcriptional profiling of renal progenitors in these various genetic mutants will likely reveal important, currently unappreciated insights about how the population is altered in the absence of these factors. Another interesting facet to this pathway is the evidence that the transcriptional coactivator Peroxisome proliferator-activated receptor gamma coactivator 1-alpha (Ppargc1a) is necessary for the development of *tbx2+* DL progenitors, and that the short DL segment in *ppargc1a^sa131186^* mutants can be rescued via *tbx2b* overexpression [143].

### 4.2. Role of Irx3b and Irx1a in DE Pronephros Development

There have been several key insights into DE segment formation in the vertebrate pronephros. Seminal work in the frog pronephros first associated several Iroquois transcription factors, including Irx1 and Irx3, with nephron segment development [144]. When assessed in the zebrafish, *irx3b* was found to be necessary and sufficient for the DE fate [77]. The knockdown of *irx3b* led to a near absence of cells expressing DE-specific markers [77]. Instead, the *irx3b*-deficient embryos had other lineages such as the CS exhibiting a population increase [77]. Furthermore, when Morales et al. examined *emx1*-deficient embryos, they observed expanded expression domains of both *irx3b* and *irx1a* in the distal tubule progenitors, suggesting that they might be a target of Emx1 and might explain the expansions in DE and CS populations in *emx1*-deficient embryos [142]. In subsequent work, Naylor et al. demonstrated that the CS lineage emerges from the DE population, thus providing a context to understand these phenotypes [145]. Additionally, prostaglandin signaling negatively regulates the *irx3b* expression domain and likely explains why interruptions in this signaling are associated with an expanded DE and reduced DL segment identity [146,147].

### 4.3. Tfap2a/b Control DE Differentiation/Maturation

**Figure 4:**
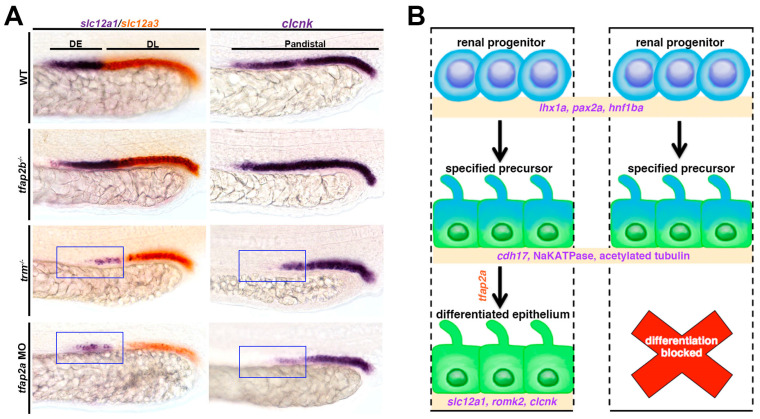
Role of Tfap2 transcription factors in DE segment differentiation in the zebrafish pronephros. (**A**) *trm* mutants exhibit reductions (indicated by blue boxes) in the expression of transcripts that encode DE segment solute transporters, such as *slc12a1*. (**B**) Tfap2a is a requisite for discrete aspects of DE differentiation, but is not required for features such as polarity establishment and ciliogenesis. Adapted from Ref. [148].

Crucial insights into the control of DE lineage differentiation came from studies on the transcription factor AP-2 alpha and AP-2 beta genes [148]. In this work, Chambers et al. isolated the *tfap2a* mutant *terminus (trm)* through a forward haploid genetic screen strategy [111] to find renal regulators by assessing the formation of alternating pronephros segments [148]. Compared to wild-type embryos, *trm* mutants showed diminished expression of the DE-specific segment marker *slc12a1* (Figure 4A). Whole genome sequencing revealed that the *trm* lesion corresponded to a G > A substitution at a conserved splice donor site in *tfap2a*. Transcript analysis revealed that mutants expressed abnormal *tfap2a* isoforms compared to wild-type embryos, including three that encoded premature stop codons. A complementation test confirmed that this novel *tfap2a* lesion causes the abnormal renal phenotype in *trm* mutant embryos, and *tfap2a* morpholino knockdown led to a similar effect (Figure 4A). Furthermore, the *trm* mutants exhibited similar abnormalities as previously reported *tfap2a* mutants in the developing neural crest, such as in the craniofacial cartilage, and these defects were rescued in overexpression studies with wild-type *tfap2a* transcripts [148]. Interestingly, both *tfap2a* and its closely related family member *tfap2b* were found to be expressed in renal progenitors [148], as noted in previous gene expression studies [136,137]. *tfap2b* loss-of-function did not alter distal segment development (Figure 4A). Interestingly, embryos that were doubly deficient for *tfap2a/2b* had the most dramatic defects in distal segment development, which requires future study. However, a loss of *tfap2a* led to diminished *tfap2b* expression, suggesting partial redundancy between the factors with *tfap2a* acting upstream of *tfap2b*. 

The severely reduced expression of DE markers in *trm* mutants but normal pattern of segment domains suggested that *tfap2a* was necessary for the terminal differential of distal nephrons (Figure 4B). *trm* mutant DE cells exhibited some differentiated features such as clearly demarcated apical-based polarity, as well as a discernible nephron lumen, together suggesting normal tubulogenesis. The cilia arrangement and morphology in *trm* mutants were also determined to be comparable with wild-type embryos, suggesting that *trm* mutant DE cells are able to undergo ciliogenesis. However, the *trm* mutant DE cells were found to have a diminished expression of specific solute transporters, leading researchers to surmise that Tfap2a was explicitly requisite for the DE to express a cadre of distal solute transporter proteins (Figure 4A). Additional genetic studies placed *tfap2a* downstream of *irx3b,* but upstream of *irx1a* in controlling DE differentiation. Taken together, these data suggest a model where *trm* mutant renal progenitor cells develop normally until a final stage of differentiation, undergoing typical specification and some aspects of differentiation such as epithelialization and organelle development (Figure 4B). However, *trm* mutant cells then fail to activate the necessary distal solute transporters in the absence of appropriate Tfap2a expression (Figure 4B). In this model, *tfap2a* is upstream of *tfap2b*. However, there is a level of redundant function that is speculated to promote the expression of the various distal solute transporter gene targets. The study does not determine whether *tfap2a* and *tfap2b* interact directly or not, which is a potential area for future investigation.

### 4.4. Tfap2a Autoregulation through the Kctd15a/b Repressors

Two candidate components of the Tfap2a nephrogenesis gene regulatory network (GRN), *kctd15a* and *kctd15b*, were hypothesized [149] because previous studies have established conserved roles for the Kctd15 potassium channel tetramerization domain genes as Tfap2 repressors [150,151]. For example, both Kctd15a/b inhibit neural crest development in zebrafish through the direct repression of Tfap2a [150,151]. Both *kctd15a* and *kctd15b* exhibited similar expressions in the distal nephron throughout pronephros development, in an overlapping pattern with *tfap2a* [149,152,153]. Loss-of-function experiments revealed that *kctd15a*, *kctd15b* and *kctd15a/b* morphants and crispants had expanded expression domains of several DE-specific differentiation markers compared to wild-type embryos [149]. The largest domains were observed in the *kctd15a/b* doubly deficient embryos, which formed nearly double the number of cells that expressed a DE signature (e.g., *slc12a1+, kcnj1a.1+*) as their wild-type counterparts [149]. Interestingly, these expansions were attributed to the co-expression of the DE signature in the adjacent proximal and distal tubule segments [149]. Furthermore, *kctd15*-deficient embryos developed a significantly expanded Corpuscles of Stannius (CS) lineage, which was derived from DE precursors [145]. These data indicate that *kctd15a/b* are required to suppress the expression of DE features, and to repress CS fate, in renal progenitors during nephrogenesis [149]. 

**Figure 5:**
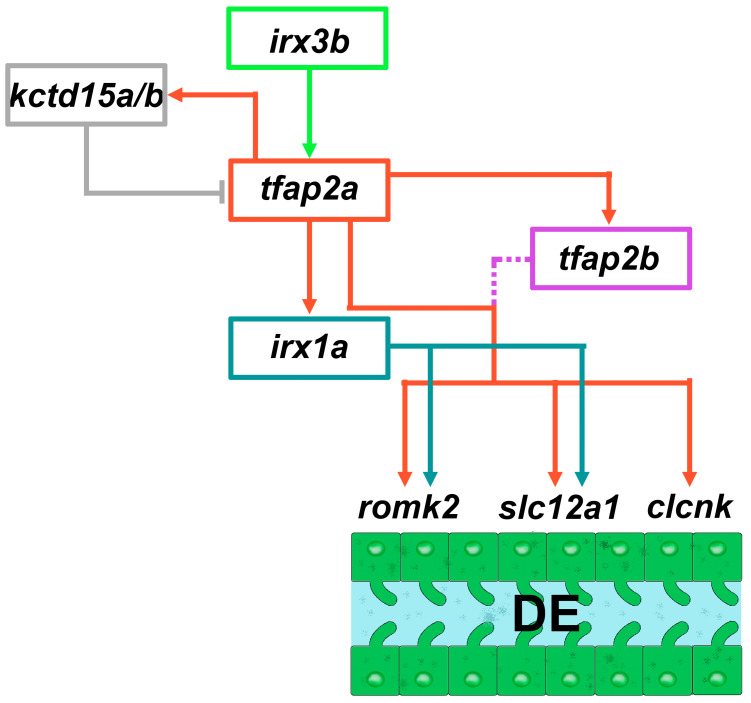
Working model of components in the Tfap2 gene regulatory network that controls distal segment differentiation during zebrafish pronephros development. *tfap2a* is positioned as an intermediary factor within a cascade of the *irx3-irx1a* transcription factors that promote DE formation. Furthermore, *tfap2a* positively regulates the expression of *tfap2b*, and auto-regulates itself by modulating the expression of the *kctd15a/b* repressors. Adapted from Refs. [148,149].

Interestingly, *kctd15a/b*-deficient embryos showed a significant expansion of the Tfap2a protein, especially in the proximal nephron [149]. These results suggest that when *kctd15a* and *kctd15b* are negated, the number of Tfap2a-expressing cells increases [149]. Indeed, the *tfap2a* expression domain was greatly increased in *kctd15a/b*-deficient embryos [149]. Furthermore, *trm* mutants exhibited significant reductions in *kctd15a/b* expression [149]. Finally, triple *tfap2a/kctd15a/b* loss-of-function studies also suggest that *kctd15a* and *kctd15b* are only able to function through *tfap2a* [149]. In summary, these findings support a model whereby an autoregulatory feedback loop driven by *tfap2a* expression regulates the transcription of its repressors, *kctd15a* and *kctd15b* (Figure 5). 

## 5. Conclusions

Here, we have discussed several critical advances in delineating the genetic factors which dictate lineage fate choice and differentiation of nephron segments in the zebrafish embryonic kidney. Ongoing work continues to identify genes of interest [154,155,156,157]. Continued investigations to elucidate the interrelationships and functions of these identified factors are an exciting future area of investigation. Additionally, data from various single cell sequencing approaches [158,159,160,161,162] are expanding our knowledge of the nephron and will be pivotal to advancing knowledge about nephrogenesis, which can be used in future applications such as regenerative medicine [163].

## Data Availability

Not applicable.
